# Calcite dissolving bacteria from peanut (*Arachis hypogaea*) pegging zone influences soil calcium level

**DOI:** 10.3389/frmbi.2022.1019134

**Published:** 2022-11-10

**Authors:** Alan Peper, Timothy Brenneman, Li Yang

**Affiliations:** ^1^ Department of Plant Pathology, University of Georgia, Athens, GA, United States; ^2^ Department of Plant Pathology, University of Georgia, Tifton, GA, United States

**Keywords:** *arachis hypogaea*, biofertilizer, calcium, calcite dissolving bacteria, soil calcium

## Abstract

Microbial communities play critical roles in mobilizing soil nutrition and, consequentially, shaping plant growth and stress responses. Soluble calcium in the pegging zone is essential for peanut yield. Calcium starvation may lead to seed abortion and increased incidence of disease, such as pod rot. Currently, gypsum or lime are often used to supplement calcium in the pegging zone. Calcite Dissolving Bacteria (CDB) can dissolve poorly soluble calcite into Ca^2+^ thus making it available to plants. Here, we report the isolation and characterization of CDB from a peanut field in Tifton, Georgia. We identified 65 CDB isolates, representing 15 unique strains belonging to 10 different genera. When applied to field soil, most of these CDB survived only several days. However, a synthetic community of CDB strains increased soluble calcium levels when applied to field soil. We also found that CDB abundancy was negatively associated with a soluble calcium level in soil. In summary, we conclude that CDB has the capacity to influence calcium availability in soil, and the abundance of CDB in a bacterial community dynamically respond to soil calcium levels.

## Introduction

The relationship between plants, microbes and nutrition is complex. Microbes can play a vital role in plant growth and stress responses (Schlaeppi and Bulgarelli, 2015; Egamberdieva and Ahmad, 2018; Saleem et al., 2019; Qu et al., 2020). For example, Plant Growth Promoting Rhizobacteria (PGPRs) are a group of rhizobacteria found in the plant rhizosphere that can promote plant growth through multiple modes of action (Ashrafuzzaman et al., 2009; Bhattacharyya and Jha, 2012; Gupta et al., 2015). The mode of action varies greatly between each PGPR, from secretion of plant hormones, secondary metabolites, volatile organic compounds, to manipulation of the abiotic environment (Vessey, 2003; Ahmed and Holmström, 2014; Kenawy et al., 2019; Riaz et al., 2021; Sukul et al., 2021). PGPRs can be developed into biofertilizers and biocontrol to support plant health and reduce the impacts of stresses. For example, phosphorus solubilizing microbes (PSMs) provide mobile forms of phosphorus by solubilizing non-soluble forms of phosphorus using organic acids, siderophores, and chelating agents (Gyaneshwar et al., 1998; Vessey, 2003; Backer et al., 2018; Kalayu, 2019).

Calcium is an essential element for plant growth. It is a limiting nutrition for peanut seed development in many peanut producing areas (Pegues et al.;Cox et al., 1976; Chen et al., 2022). As a geocarpic plant, peanut plants flower above ground, forms a peg carrying the developing embryo at its tip, and completes fruit development below ground. A developing peanut pod directly absorbs about 90% of all calcium supporting its growth from the soil, with only 10% of calcium coming from the root system (Bledsoe et al., 1949; Skelton and Shear, 1971). Soluble calcium in the geocarposphere is critical to proper pod development. Calcium starvation in the peanut pegging zone (5-7 cm below ground) during seed development may lead to aborted embryo development, compromised seed quality and eventually yield loss (Brady, 1948; Bledsoe et al., 1949; Harris, 1949; Cox et al., 1976; Yang, 2014; Chen et al., 2022). Increased incidences of soil borne fungal pathogens was also associated with calcium deficiency (Zhang et al., 2021). To ensure proper seed development and resistance to pathogens, calcium supplements in the form of gypsum (CaSO_4_) or lime (CaCO_3_) are applied yearly (Singh, 2004; Kissel and Sonon, 2008; Yang et al., 2020). For example, gypsum is suggested to be applied at a rate of 560 kg/ha (500 lbs per acre) every year at the time of flowering, and is often mandatory for seed peanuts due to its positive effects on germination (Kissel and Sonon, 2008; Eroglu et al., 2012; Pegues et al., 2017).

Soil microbes play key roles in the calcium cycle to create soluble calcium for plants to uptake. Calcinogenic bacteria can convert CO_2_ into insoluble CaCO_3_ (calcite) in presence of soluble calcium (Lüttge and Conrad, 2004). On the other hand, calcite dissolving bacteria (CDB) dissolute poorly soluble calcite to generate Ca^2+^ (Jacobson and Wu, 2009; Sulu-Gambari, 2012; Tamilselvi et al., 2016). CDB have previously been identified in calcareous soils, farmland, animal waste and limestone quarries (Subrahmanyam et al., 2009; Rana et al., 2015; Tamilselvi et al., 2016). Several mechanisms are proposed for CDB to dissolve calcite, including secreting citric acid, oxalic acid and sanazine pigment (Subrahmanyam et al., 2009; Rana et al., 2015). CDB can be selected from media containing 1% of calcite on which they form a clear zone or “halo” around the colonies (Subrahmanyam et al., 2009; Tamilselvi et al., 2016). Several commercial operations use CDB to remove calcite deposits from pipes and other unwanted calcite formations (Eroglu et al., 2012). Calcite dissolution by bacteria was also proposed as an alternative approach to reclaim calcareous sodic soils (Tamilselvi et al., 2016).

In this study, we investigated the potential role of CDB in addressing calcium deficiency in peanut field. We identified 15 CDB unique strains from a peanut growing field in Georgia, U.S. A synthetic community containing selected CDB strains increased soluble calcium level in natural soil. A negative correlation between soil calcium level and CDB abundance was observed in laboratory setting and fields. Taken together, CDB may be a key component in regulating the dynamics of calcium level in soil.

## Materials and methods

### Isolation of CDB from soil

One gram of soil collected from the peanut pegging zone in field was mixed with 9 ml of sterile water and 20 µl of cycloheximide, for fungal inhibition. After 2 minutes’ vigorous shaking, 100 µl of the soil suspension was added to a 96-well plate for serial dilution, using a factor of 1:10. The suspension was diluted to the concentration for selecting single colonies, between 1:1,000 and 1:10,000 factor dilution. 100 µl of the final dilution was spread and plated on CDB differentiating media. Plates were incubated at 28°C for 6 days. Colonies that indicate the clear zone phenotype were selected and re-isolated to obtain single CDB strains. CDB strains were then stored at -80°C.

Soil used for isolation and subsequent experiments was obtained from Blackshank Farms, Tifton GA. Five fields with various field histories and crop rotations were sampled from. Sample site histories are as below. Site 1: vegetable; site 2: peanut; site 3: peanut/cotton rotation; site 4: onion, site 5: cotton. All sample sites had similar soil texture and soil type.

The CDB differentiating media used in grams per liter was Glucose 5 g; Yeast extract 1 g; Peptone 1 g; K_2_HPO_4_ 0.4 g; MgSO_4_ 0.01 g; NaCl 5 g; (NH_4_)_2_SO_4_ 0.05 g; CaCO_3_ 5 g and Agar 7.5 g (Sulu-Gambari, 2012). The standard media used was Lysogeny broth (LB) in grams per liter Tryptone 10 g; Yeast Extract 5 g; NaCl 5 g and Agar 7.5 g.

### 16S RNA sequencing

High-Quality genomic DNA was extracted using Qaigen’s DNEASY Microbial Extraction Kit (Qiagen, Hilden, Germany). The 16s rRNA V4-V5 region of the CDB was amplified using universal primers (515F: GTGCCAGCMGCCGCGGTAA and 926R: GGACTACHVGGGTWTCTAAT) and Sanger sequenced by Eurofins Genomics (Eurofin Genomics, Louisville KY, USA 40299). The sequencing results were blasted against NCBI nucleotide collection to identify the closest hits.

### Calcite solubilization index

An overnight culture of CDB was grown in LB, pelleted, and resuspended to an OD_600 _= 0.001 at 1 ml. On a six-well culture plate (VWR, Randor PA, USA 19008), a single 10 µl droplet of CDB was placed in the center of each well containing 2 ml of CDB differentiating media. Six technical repeats were included for each CDB. The plates were incubated at 28°C. Images were taken at day 7 to measure the diameter of the colony, and the clear zone was measured through ImageJ software (NIH, Bethesda MD, USA 20814). The solubility index was calculated for each CDB using the formula $Solubility\;index=\frac{clear\;zone+colony\;size}{colony\;size}$ (Tamilselvi et al., 2016).

### Application of CDB into soil

An overnight culture of CDB grown in LB was pelleted and resuspended in 1 ml of sterile water. The suspension was then diluted to either an OD_600 _= 0.01 or 0.1 in 30 ml. The suspension was then added to 300 g of field soil with either low or high calcium level. Low soil calcium level was obtained through washing of the soil to remove Ca^2+^; low calcium ranged from 50 to 150 ppm. High calcium field soil was obtained by adding gypsum into low calcium soil, increasing soil Calcium level to 1000 ppm. Gypsum (Pennington Seed, Madison GA, USA 30650) was supplemented to achieve high calcium level. Soils with CDB were incubated 28°C for 7 days and then air dried for 3 days. Control samples were treated with 30 ml of sterile water. The samples were analyzed by the Agricultural and Environmental Services Laboratories at University of Georgia for soil pH and compositions; including Calcium, Potassium, Manganese, phosphorus, magnesium, and zinc.

### CDB competition assay

Two colonies of CDB suspensions (10ul at OD600 = 0.01) were spotted next to each other on a calcite plate. The size of targeted colonies were measured at four days after plating. The colony size from a pair of the same CDB was used as control, and its colony size was set as “1”. All members in the “TOP” mix were tested in a pairwise combination. Six repeated were performed for each combination.

### Germination test

The peanut variety used in this study was Georgia-06G. Seedlings were grown in a greenhouse (UGArden Student Community Farm, Athens GA, USA 30605) at 26°C day/23°C night, with 12 hr day/12 hr night and with 62% humidity. 30 seeds per CDB strain were soaked in a suspension of CDB diluted to OD_600_ = 0.1 at 50 ml. Seeds were soaked for 10 minutes and then air dried for 5 minutes. 10 seeds were planted in each tulip bulb pot filled with Growers Mix potting soil, and 3 pots were used for each CDB strain. Control seeds were soaked in sterile water. A randomized block design was used to arrange the pots. Seeds were grown in the greenhouse for 16 days. The percent of germination was calculated as germinated seeds/total seeds. Seedlings with two open cotyledons were considered as germinated. Data was collected 16 days after planting.

### Development of rifampicin resistant CDB strains

A dense 5 ml overnight culture of selected CDB was grown in 5 ml of LB. The culture was pelleted and resuspended in 500 µl of sterile water. 100 µl of CDB suspension was spread onto a Rifampicin 60 plate (RF_60_) (60 mg/ml) (Gold biotechnology, Olivette MO, USA 63132). Plates were incubated at 28°C for 2 days. 25 colonies were selected for each CDB and streaked onto a CDB differentiating media supplemented with Rifampicin. Mutants retaining both calcite dissolving ability and Rifampicin resistance were selected for further study.

### Survivability of CDB in soil

An overnight culture of a Rifampicin-resistant CDB strain was grown in 5 ml of LB with 5 µl of Rifampicin. The culture was pelleted and re-suspended in 1 ml of sterile water. The suspension was then diluted to an OD_600 _= 0.1 in 15 ml of sterile water. The diluted suspension was added to a 150 g of low or high calcium peanut soil. The suspension soil mixture was incubated at 28°C for 8 days. One gram of soil samples were collected every two days and the CDB were counted on RF_60_ plates after serial dilution. Ratio of CDB was determined as the proportion of CDB colonies in total culturable colonies on a LB plate.

### Soil test

Soil was oven dried (40°C), ground, and sieved through a 2-mm screen. Soil was weighed (~5.0 g), adding 20 mL Mehlich I (0.025N H_2_SO_4_ + 0.05N HCl) extracting solution. Samples were immediately placed on shaker for 5 minutes at high speed (250 oscillations per minute). Samples were then filtered using Whatman #1 paper and the extracts are analyzed for P, K, Ca, Mg, Zn, Mn, by ICP-OES (Spectro Arcos FHS16).

Soil pH and the Lime Buffer Capacity were determined using an automated LabFit AS-3000 pH Analyzer equipped with direct titration capabilities. Soil pH was determined using a 1:1 soil: 0.01 M CaCl_2_ suspension. The 0.01 M CaCl_2_ readings were then converted to soil-water pH readings by adding a conversion factor of 0.6. The Lime Buffer Capacity was determined on samples with pH readings of less than 8.1 by direct titration using 0.023 M Ca(OH)_2_.

### Statistics

Student t-test was used to compare the treated samples with controls in (**Figures 2A, C, D, E**). Student t-test was also used to compare the CDB survivability in soils with low or high concentration of Calcium. One-way ANOVA with Tukey multiple comparisons was used to compare difference treatments in (**Figures 4A, B**). Pearson correlation coefficient (r) test was used to calculate the correlation in (**Figure 5C**).

## Results

### CDB isolation and characterization

**Figure 1:**
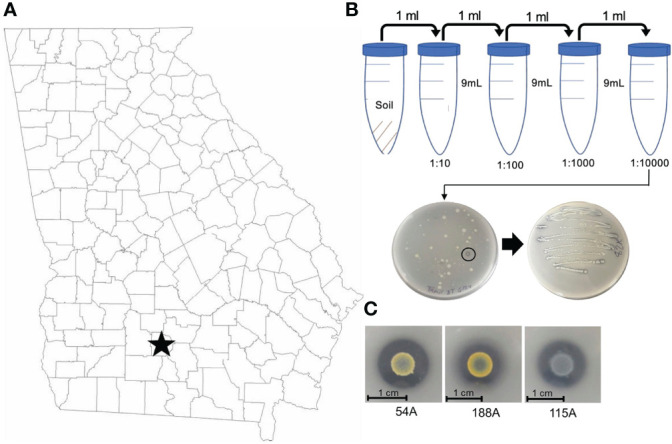
CDB isolation from peanut field in Tifton, Georgia, US. **(A)** Map of CDB survey region in Georgia, U.S. **(B)** CDB Isolation procedure from soil to single strains. Note the circled CDB colony with clear zone. **(C)** Representative CDB morphologies and images.

To identify potential CDB from a peanut producing field, we sampled the pegging zone soil from a field in continuous peanut culture at the UGA Blackshank farm (Tifton, GA) (**Figure 1A**). Soil samples were subject to a serial dilution in water. Suspensions were plated on a CDB differentiating media to visualize calcite dissolving ability of a colony. Colonies that created a clear zone were selected as potential CDB. We isolated 65 CDB strains and confirmed their ability to dissolve calcite in single colony streaks (**Figure 1B**). These CDB strains showed distinctly different colony morphologies (**Figure 1C** and **Table 1**), indicating that they belonged to different phylogenetic groups. We sequenced the 16s rRNA regions from 65 CDB colonies and identified 15 unique CDB strains. The CDB strains isolated from peanut field belongs to multiple genera including *Bacillus*, *Paenibacillus*, *Buttiauxella*, *Lelliottia*, *Cellulomonas*, *Enterobacter*, and *Staphylococcus* (**Table 1**). Multiple strains from *Bacillus*, *Paenibacillus*, *Buttiauxella* and *Lelliottia* genera were isolated. We measured the solubilization index of the 15 CDB strains on CDB differentiating plate. The solubilization index ranged from 2.1 to 5.89 (**Table 1**). We did not observe a correlation between solubilization index and bacteria genera.

**Table 1:** Characterization and identification of CDB strains.

CDB Strain ID	Genus/Species	% Identity	Solubilization Index	Colony Morphology	% Survival Rate after 8 days
17A	*Bacillus luciferensis*	98.57	2.82	Translucent, Circular, Convex, Smooth	0.88
18.2A	*Buttiauxella noackiae*	100.00	2.70	Orange, Circular, Convex, Smooth	N/A
54A	*Paenibacillus xylanexedens*	100.00	5.08	Milky white, Circular, convex, smooth	0.87
62A	*Buttiauxella warmboldiae*	99.77	2.97	Yellow, Circular, convex, smooth	N/A
70A	*Paenibacillus timonensis*	98.47	3.45	White, Circular, Convex, smooth	1.56
72A	*Enterobacter soli*	99.31	3.38	White, Circular, Convex, smooth	9.31
93A	*Bacillus megaterium*	100.00	5.17	White, Translucent, Circular, Convex, Smooth	0
95A	*Cellulomonas hominis*	100.00	4.14	White, Circular, Convex, smooth	1.57
99A	*Lelliottia aquatilis*	99.55	4.77	Translucent, Circular, Convex, Smooth	N/A
100A	*Lelliottia amnegena*	96.90	3.60	Translucent, Circular, Convex, Smooth	2.66
115A	*Buttiauxella noackiae*	100.00	5.89	White, Translucent, Circular, Convex, Smooth	6.19
130.2A	*Bacillus circulans*	98.87	2.10	Milky, Circular, Convex, smooth	N/A
140A	*Paenibacillus etheri*	100.00	2.33	Translucent, Circular, Convex, Smooth	N/A
141A	*Paenibacillus phocaensis*	100.00	2.88	Translucent, Circular, Convex, Smooth	N/A
188A	*Staphylococcus pasteuri*	99.55	5.89	Orange, Circular, Concave, Smooth	N/A

### Application of CDB alters soil calcium level

**Figure 2:**
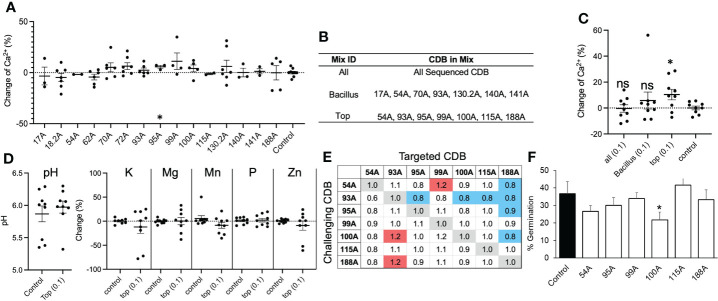
CDB alters soil calcium availability. **(A)** Percent change in soil calcium level after application of single strain CDB at OD_600_=0.01. Water was used as a control. * above 95A indicate *p*< 0.05 when compared to control using student t-test. **(B)** Table listing components of each synthetic CDB community. **(C)** Soil calcium level after applying synthetic CDB communities. Each dot represents an independent test. Long bars and short bars represent means and standard errors, respectively. * indicates *p<* 0.05 when compared to control using student t-test. ns: not significant. **(D)** Soil pH and nutrient level after applying the “TOP” mix. **(E)** Pairwise interaction between members in the “TOP” CDB mix. The numbers indicate normalized colony size of targeted CDB. The targeted CDB colony size was normalized to “1” when the same bacterium was used as challenging CDB. Red and blue shade indicate significant promoting and inhibiting effect based on a comparison of colony size using student t-test. **(F)** Germination percent of Georgia-06G after seed soaking with single strain CDB suspension. * indicates *p* < 0.05 when compared to control using student t-test.

To test if adding CDB in soil can increase soluble calcium level, we collected field soil samples from the same location where CDB were isolated. The average calcium concentration was around 200 ppm. Soils were supplemented with individual CDB strains at OD_600 =_ 0.1 in laboratory and incubated in 28°C incubator. Increased calcium level was observed in many cases after adding individual CDB (**Figure 2A**). However, extensive variations were observed between repeats, despite our efforts to standardize the protocol. Only one CDB (95A) significantly increased calcium level. We reasoned that individual CDB had limited capacity to influence soil calcium level, and the heterogeneity of natural soil increased the variation between repeats.

To improve the consistency of CDB application, we generated three synthetic communities of CDB. The “TOP” mixture contained 7 CDB strains that showed the highest solubilization index on plate (**Figure 2B**); the “ALL” mixture contained all 15 CDB strains; and the “Bacillus” mixture contains seven *Bacillus* and *Paenibacillus* CDB strains (**Figure 2B**). We chose *Bacillus*/*Paenibacillus*, because they can generate subtilis spore, which may increase survivability of CDB in soil. When applied at OD_600 _= 0.01, none of these mixtures changed the level of calcium in soil. At a high concentration (OD_600 _= 0.1), the “TOP” mixture significantly increased calcium level (**Figure 2C**). Interestingly, soil pH was not changed by the “TOP” mixture as well as the levels of Potassium, Manganese, phosphorus, magnesium, and zinc (**Figure 2D**). To explore the potential interaction between members of the “TOP” mix, we performed a competition assay to compare CDB multiplication in a pairwise combination on calcite plates (**Figure 2E**). We observed a complex interaction pattern between “TOP” CDB members. Mainly, 93A inhibited the colony growth of 4 out of 6 “TOP” community members, and 188A multiplication was inhibited by 4 members (54A, 93A, 95A and 100A), indicating potential interactions between these CDBs in soil (**Figure 2E**). We also found that CDB strains included in the “TOP” mix were not pathogenic, although one strain (100A) slightly reduced germination rate (**Figure 2F**). These results suggested that supplementing CDB could increase soluble calcium level in natural soil.

### Survival rate of CDB in field soil

**Figure 3:**
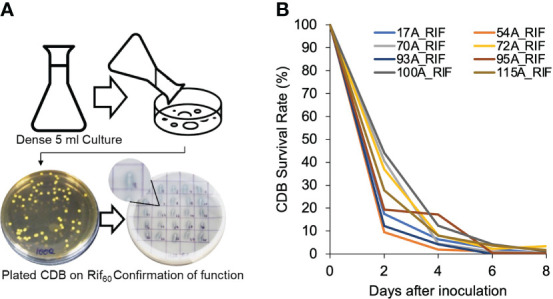
Supplemented CDB have short life in soil. **(A)** Procedure to create rifampicin resistant CDB mutants and confirmation of calcite dissolving phenotype. In the CDB differentiation plate, note the clear zone created by CDB strains recovered from Rif_60_ plates. **(B)** CDB-RIF survival rate in soil. CDB-RIF was added at a concentration of OD_600_=0.1. Survival rate was determined by comparing the colony forming unit of CDB recovered from a Rif_60_ at each day to that of Day 0. Measurements was taken every two days until day 8.

We hypothesized that the variation of individual CDB’s impact on calcium level was due to the short survival time of supplemented CDB. To monitor the survival rate of supplemented CDB in soil, we generated mutants of CDB that are resistance to Rifampicin (CDB-RIF) (**Figure 3A**). Importantly, the Rif-resistance CDB still have the capacity to dissolve calcite (**Figure 3A**). We added individual Rif-resistant CDB mutant into soil and measured the amount of CDB that could be recovered on a Rifampicin plate. For the tested 8 CDB strains, their abundance dropped to less than 30% after the first two days. 95A-Rif maintained a higher survival rate compared to other CDB at day 4, which was consistent with its impact on soil calcium level when added individually (**Figure 2A, B**). One week after application, added CDB were not detectable (**Figure 3B**). Thus, supplemented CDB have short survival time even in native soil.

### Correlation between CDB abundance and soil calcium level

**Figure 4:**
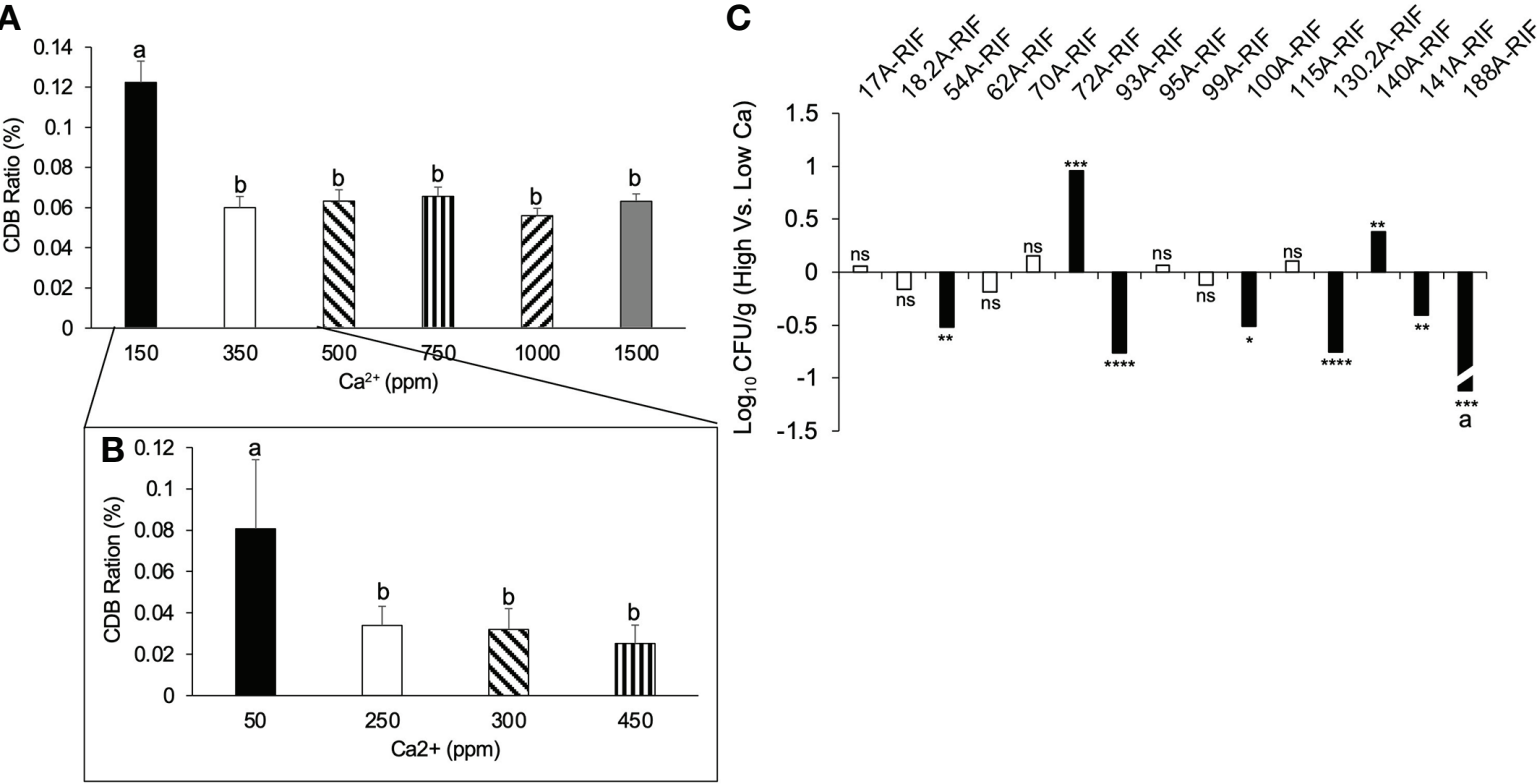
Correlation between CDB abundance and soil calcium level. **(A)** Relative CDB abundance in soils supplemented with different concentration of calcium. error bars: standard error; different letters on top of each bar represent significant difference using One way ANOVA with a Tukey multiple comparison. Calcium was supplemented in the form of gypsum. **(B)** Relative CDB abundance responding to a fine scale of calcium concentration. CDB ratio was determined by dividing the number of CDB colonies to the number of total culturable colonies on CDB differentiation plate. **(C)** Individual CDB response to soil calcium level. *p < 0.05; **p < 0.01; ns: not significant. Student t-test was used to compare the abundance of CDB-RIF (Log(CFU/g)) from high and low calcium soil. a: 188A-RIF was only recovered from low calcium soil. ***p < 0.001; ****p < 0.0001.

To test the relationship between CDB abundance and soil Ca^2+^ level, we monitored the relative abundance of CDB in low calcium field soil supplemented with different levels of gypsum. The relative abundance of CDB was determined by the ratio of CDB colonies to the total number of culturable colonies on a calcite plate. We found a reduction of CDB proportion when Ca^2+^ concentration increased from 0 to 350 ppm (**Figure 4A**). No further reduction was observed when Ca^2+^ level exceeded a threshold of 350 ppm, indicating that the increase of CDB level in low calcium soil might be an adaptation to calcium deficiency. We further narrowed the threshold concentration to 250 ppm (**Figure 4B**). It is noteworthy that the calcium concentration in untreated field soil was around 200 ppm, which is close to the threshold. To directly observe CDB’s response to calcium level and test which CDB contributed to this overall reduction of CDB population in response to increased calcium level, we inoculated a high (1000 ppm) and low (100 ppm) calcium soil with individual Rifampicin-resistant CDB strains (**Figure 4C**). Two days after inoculation, we compared the abundance of inoculated CDB on a Rif_60_ plate. Two of the 15 tested strains showed increased survivability in high calcium soil. 54A-RIF, 93A-RIF, 100A-RIF, 130.2-RIF, 141A-RIF and 188A-RIF strains had decreased survivability in high calcium soil (**Figure 4C**). It is noteworthy that we were not able to recover 188A-RIF from high calcium soil two days after inoculation. These results indicate that individual CDB strains have distinct response to soil calcium level, and a few CDB strains may contribute to the observed overall negative correlation between CDB abundance and calcium level.

**Figure 5:**
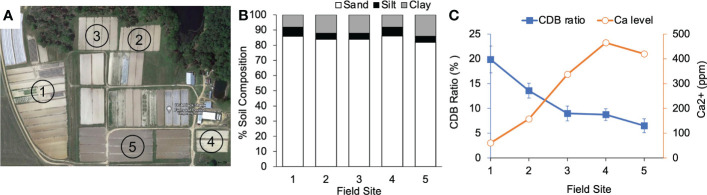
CDB population response to calcium in field soil. **(A)** Arial photo of sample sites in Blackshank Farm, Tifton, GA. Numbers in each field correspond to numbers on X axis in **(B, C)**. **(B)** Soil texture analysis of the 5 field sample sites. **(C)** A negative correlation between calcium level in soil and relative abundance of CDB within field soils. CDB ratio was determined by dividing the number of CDB colonies to the number of total culturable colonies on CDB differentiation plate. 10-15 plates were used for each soil sample.

To test if the negative correlation between CDB abundance and soil calcium level occurs in nature, we sampled soils from five fields in the Blackshank farm (**Figure 5A**). The field soils had similar structure with 85% sand and 15% silt and clay (**Figure 5B**). We observed that soils from different fields contained variable about of Ca^2+^ (**Figure 5C**). Field 3 and 4 had the highest calcium level and the lowest CDB ratio (**Figure 5C**). There was a negative relationship between relative CDB abundance and soil Ca^2+^ level in these samples (Pearson correlation coefficient (r= -0.9352)). The negative relationship was consistent with what we observed from soils supplemented with different amounts of gypsum in lab (**Figure 4A**). Taken together, our observations indicated that the soil CDB abundance decreased as a response to elevated soil calcium level.

## Discussion

Calcium is a well-known limiting nutrient in peanut production and has been linked to disease severity from soil borne fungal pathogens i.e., *Aspergillus flavus (A. flavus)* (Fernandez et al., 1997; Yang, 2014; Yang et al., 2017). In this study, we isolated CDB strains from a peanut growing field in Georgia, USA, representing species from different genera. Previously, CDB were identified from limestone quarries and animal fecal matter (Tamilselvi et al., 2016). Other research has shown CDB to be an effective means to solubilize calcite and have used them to reverse the desertification process, restore statues, and remove calcium build-up from pipes (Eroglu et al., 2012; Rana et al., 2015; Anbu et al., 2016; Tamilselvi et al., 2016; Qu et al., 2020). Interestingly, CDB isolated from these studies and our research belong to distinct phylogenic groups, indicating that different bacteria with calcite dissolving ability adapted to these environments.

The identification of CDB in peanut fields provides the potential for a deeper look into the peanut microbiome as well as a start to further investigate the microbial impact on the soil calcium cycle. As a geocarpic plant, peanut fruits share the same environment as their root. Their roots and pods associate with distinct and overlapping microbiomes, presumably cooperating with different physiological needs of these two organs (Bledsoe et al., 1949; Harris, 1949; Kvien et al., 1988; Smal et al., 1989). Pod-associated microbiota was shown to affect *A. flavus* infection and the production of aflatoxin (Chourasia and Sah, 2017; Zhang et al., 2021). Selected geocarposphere bacteria have potential as a biocontrol for soil borne fungal pathogens such as *Aspergillus* (Chourasia and Sah, 2017; Li et al., 2020; Zhang et al., 2021). Since peanut pods actively absorb a large amount of calcium to support embryo and shell development, it is intriguing to know whether CDB are differentially associated with pods and roots and whether they can promote pod development.

We found that single CDB strains had limited impact on the calcium level in natural soil (**Figure 2A**). Only one strain, 95A, significantly increased calcium level (**Figure 2A**). However, a synthetic community composed of CDB with high solubilization index significantly increased soluble calcium level (**Figure 2C**). It is likely that a single CDB strain is not competitive in the soil microbial community, leading to a short survival time as indicated by our CDB survival study. The ability of “TOP” synthetic community to increase calcium level implies that the solubilization index on plate could be an indicator of calcite dissolving ability in soil. It is also possible that the “TOP” synthetic community increases the survivability of members in soil. To develop CDB as a biofertilizer, it is a future challenge to further increase the potency of CDB community in soil. One approach is to identify the core microbiome associated with the peanut rhizosphere and geocarposphere. Core microbiomes have been used to stabilize synthetic communities (Armanhi et al., 2018; Toju et al., 2018; Bano et al., 2021; Fazeli-Nasab et al., 2022). Further investigation using tagged bacteria will help to track CDB in a complex soil environment and understand the mechanism underlying their low survivability in high calcium soils. Alternatively, CDB may be applied *via* other methods such as a seed coating or even an in-furrow spray (Malusá et al., 2012; Herrmann and Lesueur, 2013; Ma, 2019; Agake et al., 2021). One of the greatest threats to peanut production is the increased presence of *A. flavus* and its potential to produce the mycotoxin aflatoxin. Studies showed that *A. flavus* infection on peanut and aflatoxin accumulation was negatively correlated with soil calcium level (Zhang et al., 2021). Thus, CDB may also be used as an alternative method to control *A. flavus.*

## Conclusion

Our results show that CDB are present in Georgia peanut fields and can be successfully isolated. Individual CDB strains showed limited capacity to influence soil soluble calcium level, probably due to short survival time. However, a logically design synthetic community increased calcium level in natural soil. The relative abundance of CDB in soil was negatively correlated with calcium level. Our study suggested the potential of CDB to engineer soil calcium availability for peanut production.

## Data availability statement

The original contributions presented in the study are included in the article/Supplementary Material. Further inquiries can be directed to the corresponding author.

## Author contributions

All authors contributed to the article and approved the submitted version.

## Funding

This research was funded by the University of Georgia seed grant and Georgia Peanut Commodity Commission grant UGA48-19/21.

## Acknowledgments

We thank University of Georgia county extension coordinators Brian Hayes and Bill Starr for assistance with soil sampling. Drs. Soraya Bertioli, Albert K. Culbreath and Cristiane Pilon for helpful discussion on this project. We thank the Agricultural and Environmental Services Laboratories at University of Georgia for providing soil analysis.

## Conflict of interest

The authors declare that the research was conducted in the absence of any commercial or financial relationships that could be construed as a potential conflict of interest.

## Publisher’s note

All claims expressed in this article are solely those of the authors and do not necessarily represent those of their affiliated organizations, or those of the publisher, the editors and the reviewers. Any product that may be evaluated in this article, or claim that may be made by its manufacturer, is not guaranteed or endorsed by the publisher.
